# Behavioral Response in the Immediate Aftermath of Shaking: Earthquakes in Christchurch and Wellington, New Zealand, and Hitachi, Japan

**DOI:** 10.3390/ijerph13111137

**Published:** 2016-11-15

**Authors:** Ihnji Jon, Michael K. Lindell, Carla S. Prater, Shih-Kai Huang, Hao-Che Wu, David M. Johnston, Julia S. Becker, Hideyuki Shiroshita, Emma E.H. Doyle, Sally H. Potter, John McClure, Emily Lambie

**Affiliations:** 1 Department of Urban Design and Planning, University of Washington, Seattle, WA 98195, USA; mlindell@uw.edu (M.K.L.); csprater@uw.edu (C.S.P.); 2 Department of Emergency Management, Jacksonville State University, Jacksonville, AL 36265, USA; skysnow0080@jsu.edu; 3 Department of Political Science, Oklahoma State University, Oklahoma City, OK 73107, USA; tristan.wu@okstate.edu; 4 Joint Centre for Disaster Research, GNS Science/Massey University, Wellington 6140, New Zealand; David.Johnston@gns.cri.nz (D.M.J.); J.Becker@gns.cri.nz (J.S.B.); E.E.Hudson-Doyle@massey.ac.nz (E.E.H.D.); s.potter@gns.cri.nz (S.H.P.); 5 Faculty of Safety Science, Kansai University, Suita-shi, Osaka 564-8680, Japan; hideyuki@kansai-u.ac.jp; 6 School of Psychology, Victoria University of Wellington, Wellington 6140, New Zealand; John.McClure@vuw.ac.nz; 7 Department of Geological Sciences, University of Canterbury, Christchurch 8140, New Zealand; emily.lambie@pg.canterbury.ac.nz

**Keywords:** earthquakes, post-impact response actions, risk perception

## Abstract

This study examines people’s response actions in the first 30 min after shaking stopped following earthquakes in Christchurch and Wellington, New Zealand, and Hitachi, Japan. Data collected from 257 respondents in Christchurch, 332 respondents in Hitachi, and 204 respondents in Wellington revealed notable similarities in some response actions immediately after the shaking stopped. In all four events, people were most likely to contact family members and seek additional information about the situation. However, there were notable differences among events in the frequency of resuming previous activities. Actions taken in the first 30 min were weakly related to: demographic variables, earthquake experience, contextual variables, and actions taken during the shaking, but were significantly related to perceived shaking intensity, risk perception and affective responses to the shaking, and damage/infrastructure disruption. These results have important implications for future research and practice because they identify promising avenues for emergency managers to communicate seismic risks and appropriate responses to risk area populations.

## 1. Introduction

In the immediate aftermath of an earthquake, many victims’ survival and injury outcomes depend on the response of others who are in the impact area. Conversely, many of those who have survived unscathed must choose whether to resume their previous activities, seek information—about the situation generally or about their loved ones more specifically—or try to cope with the disaster by cleaning up debris or helping other people. It is well known that volunteers and emergent groups are a major source of early search and rescue (SAR) activity [1]. Earthquake epidemiological research has provided data about the proportion of trapped victims who are rescued by untrained uninjured victims; however, little is known about the proportion of bystanders who take action after the shaking stops to engage in SAR—let alone other activities. Therefore, to better understand how people behave in the immediate aftermath of earthquakes, this study examines people’s actions during the first 30 min after four earthquakes in New Zealand and Japan. The following sections (1) review the social science literature on people’s actions in the immediate aftermath of earthquake shaking, (2) propose eight research questions and three research hypotheses, (3) describe the methods by which the present study was conducted, (4) present the survey results, and (5) discuss the theoretical and practical implications of those results.

## 2. Literature Review

### 2.1. Previous Research

There has been a significant amount of research on household preparedness for earthquakes [2,3,4,5,6,7,8,9,10,11] but there has been substantially less research on people’s responses during earthquakes. Recent studies by Prati et al. [12] and Lindell et al. [13] have built on previous work [14,15,16,17,18,19,20]. Specifically, the Prati et al. [12] study of the 2012 Emilia-Romagna MW 6.1 earthquake found that the most common response was to move to another room (42%), followed by evacuating the home (36%), waiting in bed (33%), going downstairs (28%), getting dressed (19%), sheltering in a doorway (14%), sheltering near a supporting wall (14%), and sheltering under a table (2%). There were no significant predictors of the three sheltering behaviors, probably because they were so infrequent, and fear was the most consistent predictor of all five other behaviors. The Lindell et al. [13] study of the 2011 Christchurch and Tohoku earthquakes reported that the most common response during the shaking was to freeze in place (34%), followed by evacuate immediately (20%), duck, cover and hold on (12%), protect persons (8%), protect property (8%), and continue normal activities (2%). Logistic regression analysis revealed inconsistent predictors for the three most common responses—freezing, evacuating, and covering.

There is even less information about people’s behavior in the immediate aftermath of earthquake shaking although SAR activity has been a major focus of most accounts. For example, Noji [21] reported that 90% of the victims in an Italian earthquake were rescued by untrained volunteers. Wenger [22] indicated that participation in SAR activity is related to location (i.e., impact proximity), knowledge about the safety of significant others, identification with the community, emergency-relevant training, and membership in emergency-relevant organizations. Additionally, Friedsam [23] concluded that rendering assistance to others beyond the family was negatively related to age, and Form and Nosow [24] concluded rendering help to strangers was more likely among men than women. However, Takuma’s [20] report of people’s actions after the 1968 Ebino earthquake provides no mention of helping others. Instead, 24% of victims sought refuge, 18% turned off fuel outlets, 11% prepared to seek refuge, 8% sought information, 7% entered the house (39% had sought refuge outdoors), 7% “did nothing” (presumably resuming previous activities), and 5% located items for self protection or put things back in order. Thus, the research literature has produced rather disparate accounts of the extent to which untrained uninjured victims are actively involved in SAR activities—let alone other actions in the immediate aftermath of earthquakes.

### 2.2. Theoretical Framework

One limitation of previous research on immediate aftermath actions is the lack of a theoretical framework. This need can be filled by Lindell and Perry’s [25,26,27] Protective Action Decision Model (PADM), which summarizes research explaining how the social and environmental context affects environmental cues, social cues, and socially transmitted warnings. In turn, these produce perceptions of threat, alternative protective actions, and social stakeholders that also depend on people’s personal characteristics (past experience, personal resources, and demographic characteristics). These, in turn, produce information search strategies and protective action decision making that, depending on the presence of facilitating conditions or impediments in the situation, result in behavioral responses.

### 2.3. Research Questions and Hypotheses

To date, most research relevant to the PADM has focused on pre-impact protective actions, especially recommendations to evacuate from hurricanes or shelter in-place from tornadoes. However, there are many different types of actions that might be taken in the immediate aftermath of an earthquake, such as information seeking and actions to protect persons and property. Consequently, it is important to assess the relative prevalence of each type of action. In addition, the choice of different post-impact actions would be expected to depend on the variables identified by the PADM—social and environmental context, environmental cues, social cues, socially transmitted warnings, and personal characteristics such as past experience, personal resources, and demographic characteristics. These considerations lead to eight research questions and three research hypotheses about the prevalence of different post-impact actions and the variables that might explain them.

RQ1. How did behavior during the immediate aftermath (i.e., the next 30 min after shaking stopped) differ across earthquakes?
RQ2. Do demographic variables, such as age, gender, and marital status, affect peoples’ behavior during the immediate aftermath?
RQ3. Does earthquake experience and emergency preparedness affect behavior during the immediate aftermath?
RQ4. Does physical context, such as being at home, affect behavior during the immediate aftermath?
RQ5. Does household context, such as the location of family members, affect behavior during the immediate aftermath?
RQ6. Does social context, such as whether the individual is with others, affect behavior during the immediate aftermath?
RQ7. Do actions taken during shaking affect behavior during the immediate aftermath?
RQ8. What communication channels were used to seek additional information about the situation and to contact separated household and/or family members?

There is a common tendency for people to label disaster victims as disorganized or panicked and in need of economic, social, and emotional support. Moreover, this tendency usually assumes that such support can only be effectively organized by external actors or organization. Despite these popular stereotypes, however, disaster research conducted since the 1960s has demonstrated that those affected do not merely remain passive “victims,” but rather become active participants in emergent organizations [28,29,30,31,32]. Thus, people will respond to earthquake shaking, home damage, and infrastructure interruption by taking adaptive action.

RH1. Perceived shaking intensity, risk perception and affective response are negatively correlated with returning to previous activities, but positively correlated with behaviors such as turning off utilities, contacting household members, cleaning up, and helping others.
RH2. Home damage and infrastructure interruption are negatively correlated with returning to previous activities, but positively correlated with behaviors such as turning off utilities, contacting household members, cleaning up, and helping others.
RH3. Injuries are positively correlated with helping others but negatively correlated with the other immediate aftermath actions.

## 3. Method

### 3.1. Settings

**Figure 1:**
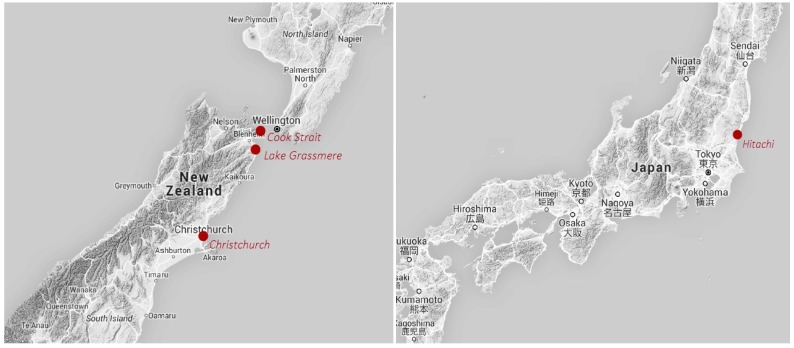
Earthquake survey locations.

The 2011 Christchurch earthquake was a M_W_ 6.3 event that struck at 12:51 local time on 22 February, causing 185 deaths, some 7500 injuries, and USD 11 billion of damage [5,33]—see Figure 1. The city of Christchurch, which experienced 12 s of severe shaking (a maximum of MMI IX), was the area most severely affected by the earthquake. The 2011 Tōhoku M_W_ 9.0 earthquake struck Japan at 14:46 local time on 11 March. The 3−5 min of severe earthquake shaking (a maximum of MMI VII in Hitachi) and resulting tsunami caused 15,854 deaths, 26,992 injuries, and USD 235 billion of damage throughout northeast Japan. Two years later, in July and August 2013, two earthquakes occurred near Wellington, New Zealand that were widely felt and injured over 100 people. The Cook Strait earthquake, which occurred at the depth of 13 km about 51 km from central Wellington, was a M_W_ 6.5 event that struck at 17:09 and lasted for 7.5 s, producing a maximum of MMI VI in Wellington. The Lake Grassmere earthquake, which occurred at a depth of 8 km about 77 km from central Wellington, was a M_W_ 6.6 event that struck at 14:31 and lasted for 2–3 s, producing a maximum of MMI VI in Wellington. In combination, the 2013 earthquakes caused NZ$ 30 million of insured earthquake damage to residential properties. Although the Tōhoku event had a much greater magnitude than the other three events, the sample of Hitachi residents selected for that event was located much farther away from the earthquake epicenter than the Christchurch or Wellington samples were from their respective epicenters. The local intensities were thus more similar than the source magnitudes.

### 3.2. Samples

Following the 2011 Christchurch and Tōhoku earthquakes, researchers from Texas A&M University drafted a questionnaire in collaboration with colleagues from New Zealand (GNS Science/Massey University, Wellington, New Zealand) and Japan (Kansai University, Osaka, Japan)—see Lindell et al. [13] for details. The study team then selected a random sample of 600 households from census (Christchurch) or voter registration (Hitachi) records. Each household received an initial notification letter with a questionnaire, a reminder postcard, and a follow-up questionnaire if they did not return a completed questionnaire. The Christchurch data were collected from September–December 2011 and the Hitachi data were collected from January—April 2012. The procedure yielded 257 (42.8%) usable questionnaires from Christchurch and another 332 (55.3%) from Hitachi.

Following the 2013 Cook Strait and Lake Grassmere earthquakes, Fraser et al. [34] used the same questionnaire to collect data in six coastal suburbs of Wellington (Island Bay, Lyall Bay, Owhiro Bay, Houghton Bay, Melrose, and Southgate). A total of 600 questionnaires were hand-delivered in October 2013 to randomly selected residences, and 404 questionnaires were mailed to those who did not respond to the initial delivery. This procedure yielded 204 (34%) usable questionnaires.

### 3.3. Measures

The questionnaire asked respondents to report the community in which they were located at the time of the earthquake, their physical context (own home, home of friends or family, workplace, public place such as shopping or recreation facility, driving a vehicle, passenger in a vehicle, other), social context (alone, with children under 18 years of age, with known adults, with adult strangers), and household context (all household members together, some household members absent but known to be in a safe location, some household members absent and known to be in danger, some household members absent and unknown if they were safe). Respondents were also asked to report their perceptions of shaking intensity (Not felt = 1, Weak shaking = 2, Mild shaking = 3, Moderate shaking = 4, Strong shaking = 5, Violent shaking = 6).

Respondents were next asked to indicate which one action best described their first behavioral response during the shaking: continued what they were doing before the shaking; stopped what they were doing but stayed where they were; dropped, covered under a sturdy piece of furniture such as a table or desk, and held on to it; tried to protect other people nearby; tried to protect property nearby, such as preventing things from falling; immediately left the building they were in; continued driving; pulled over to the side of the road; or other. Each action was coded No = 0 or Yes = 1.

Consistent with the Protective Action Decision Model, people’s perceptions of the earthquake threat were assessed by the perceived likelihood of four personal consequences: damage to their homes; injury or death to themselves and their loved ones; disruption of their jobs; and a loss of utilities. The four items were rated on a scale from Not at all = 1 to A very great extent = 5 and summed to form a scale with α = 0.72. Respondents also reported their affective reactions to earthquake shaking on a nine-item subset of the UWIST MAC (University of Wales Institute of Science and Technology Mood Adjective Checklist), each item being measured on a scale from Not at all = 1 to A very great extent = 5. Three items each measured shock (depressed, annoyed, passive; α = 0.70), fear (alert, nervous, fear; α = 0.75), and vigilance (optimistic, relaxed, energetic; α = 0.59).

Next, respondents reported what they did in the first 30 min after the shaking stopped. The response options were: returned to what I was doing before the shaking, tried to find out what had happened, turned off utilities (gas, electricity, or water), tried to contact household members, cleaned up broken or fallen items, helped people near me, went to a clinic or hospital for treatment, went to my home, went to the home of a friend or relative, went to a public shelter, went somewhere else, and other. Respondents were allowed to check all actions they took and each was coded No = 0 or Yes = 1. In addition, they reported the communication channels they used during that time: none, face-to-face conversation, television, radio, telephone, and Internet (asked only in the Wellington events), with each coded No = 0 or Yes = 1.

Respondents were asked to report their earthquake experience prior to the one being studied in terms of four items: whether an earthquake had damaged property in their community, damaged their homes, injured or killed a member of their family, or injured themselves (No = 0, Yes = 1). These four items were averaged to form a scale with α = 0.55. They were also asked to indicate if they had, previous to the earthquake being studied, obtained earthquake information by attending a meeting on the earthquake hazard or receiving a brochure about earthquake hazard (No = 0, Yes = 1). These two items were averaged to form a scale with α = 0.37. Next, they were asked to report their level of household emergency preparedness by indicating whether or not they had a three-day supply of water, a three-day supply of non-perishable food, an emergency kit filled with supplies, a household emergency plan, a battery-powered radio, and a predetermined place to evacuate (No = 0, Yes = 1). These six items were averaged to form a scale with α = 0.63. In addition, they were asked to report whether anyone in their household was killed or injured, how much damage their home experienced (none, slight, moderate, severe, totally destroyed), and what types of infrastructure were interrupted (electric power, water, sewer, Internet access, satellite or cable TV access, telephone, gas)—each coded No = 0 or Yes = 1. The seven infrastructure interruption items were averaged to form a scale with α = 0.91.

Finally, respondents were asked to report nine demographic characteristics: age; gender; marital status (married, single, divorced, or widowed); the number of people in their household under 18 years, between 18 and 65 years, and over 65 years; whether anyone in their household had a disability requiring assistance; and how long they had lived in the community where they now resided. In Christchurch and Wellington, the respondent’s education level was categorized: no school qualifications; school qualifications such as trade certificate, professional certificate, or diploma; university undergraduate degree (such as university diploma or bachelor’s degree); or university postgraduate degree (such as a Master’s or a Ph.D.). In Hitachi, the response options were: less than ninth grade; ninth to twelfth grade, no diploma; high-school graduate (includes equivalency); some college, no degree; associate degree; bachelor’s degree; or graduate or professional degree. Yearly household income was categorized as less than USD 20,000, between USD 20,000 and 29,999, USD 30,000 and 39,999, or USD 40,000 and 49,999, or USD 50,000 or more, and was translated into the corresponding currency (NZD or JPY) at the prevailing exchange rate. Finally, respondents reported whether they owned or rented the home in which they lived.

### 3.4. Analyses

As in Huang et al. [35], the equivalence of the patterns of intercorrelations among the responses to the questionnaire items between communities was assessed by taking the obtained value of each correlation for respondents from one event and plotting it against the corresponding value of that correlation for respondents from another event. For example, one data point is defined by plotting the value of the correlation between gender and perceived shaking intensity for Christchurch on the x-axis and the corresponding value of the correlation between gender and perceived shaking intensity for Hitachi on the y-axis. Thus, the total number of data points is equal to the distinct correlation coefficients in the correlation matrix for each of the four events—k (k−1)/2 = 40 (39)/2 = 780. This procedure yielded six cross-plots that were approximately linear and had no obvious outliers, indicating a similar overall pattern of intercorrelations among the questionnaire items in the four events. Consequently, a weighted-average correlation matrix was created by multiplying the correlation in each earthquake by the sample size for that earthquake, adding the weighted correlations, and dividing by the total sample size for all four earthquakes. The data were analyzed using IBM Statistical Package for the Social Sciences, SPSS (IBM SPSS Inc., Chicago, IL, USA), and R open software (R Studio Inc., Boston, MA, USA).

One potential consequence of the large number of correlations is an inflated experiment-wise error rate. Specifically, one could expect that there would be approximately 39 coefficients that are classified as “statistically significant” at the 5% level just by chance alone. Consequently, the results presented below only list correlations as statistically significant if *p* ≤ 0.001. Thus, one can expect only one correlation coefficient being considered “statistically significant” just by chance alone. Moreover, the large sample size (N = 793) allows even *r* = 0.11 to be classified as statistically significant even though this accounts for only about 1% of the shared variance. Consequently, only *r* ≥ 0.15 are discussed below.

## 4. Results

Table 1 shows the demographic characteristics of the Christchurch, Hitachi and Wellington samples in comparison to the census data for those cities. The Christchurch sample over-represents residents who are older, married, home-owning, more highly educated, female, and have higher incomes. The Hitachi sample over-represents residents who are older, married, home-owning, and more highly educated, but accurately represents income and slightly under-represents females. The Wellington sample over-represents residents who are older, married, home-owning, more highly educated, and have higher incomes but accurately represents females.

**Figure 2:**
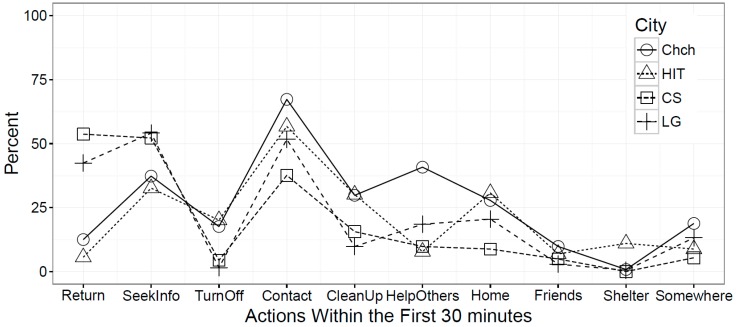
Percentage of response actions within 30 min after shaking caused by earthquake stopped.

Across the four earthquakes, respondents’ most frequent activity during the 30 min after shaking stopped was to contact household members (54.3%), followed by seeking further information (42.3%) and resuming previous activities (24.8%). Relatively few people turned off utilities, cleaned up broken or fallen items, helped others, or went elsewhere (clinic/hospital, own home, friend/relative home, public shelter, or other location). However, Figure 2 shows that, regarding RQ1 (How did behavior during the immediate aftermath after shaking stopped differ across earthquakes?), some activities (resume previous activities, seek information, turn off utilities) reveal a contrast between the Christchurch and Hitachi events versus the two Wellington events (Cook Strait/Lake Grassmere). Christchurch (12.5%) and Hitachi (5.6%) had few people who returned to what they were doing before the earthquake, compared to Wellington where almost half of the affected populations (Cook Strait: 53.7%, Lake Grassmere: 42.4%) resumed previous activities. Another difference is that more people turned off their utilities and were involved in cleanup activities in Christchurch and Hitachi than after the two Wellington events. Moreover, Christchurch had a much higher percentage involved in helping others (40.8%) than Hitachi (7.8%), Cook Strait (9.8%), and Lake Grassmere (18.5%).

Regarding RQ2 (Do demographic variables affect behavior during the immediate aftermath?), Table 1 shows that age was the only variable with a significant correlation. Older respondents were less likely to contact household members. Regarding RQ3 (Do earthquake experience and emergency preparedness affect behavior during the immediate aftermath?), Table 2 shows that the pre-impact variables had no statistically significant correlations with immediate aftermath actions.

As for RQ4 (Does physical context—being at home—affect behavior during the immediate aftermath?), the results showed that people who were in their own home at the time of earthquake were less likely to contact household members. This result can be explained by the fact that respondents who were at home were highly likely to be together with other household members at the time of the earthquake (*r* = 0.75).

The data related to RQ5 (Does household context affect behavior during the immediate aftermath?) reveal that household context did influence people’s actions immediately after the shaking stopped. Respondents who were together with household members were less likely to contact household members; respondents whose household members were present or known to be safe were less likely to contact household members, whereas those with family members absent and safety unknown were more likely to do so.

Answering RQ6 (Does social context affect behavior during the immediate aftermath?), the results indicate that the social context (whether they were alone, or with children, or with adults) did not seem to affect people’s activities immediately after the shaking stopped. The percentages of people who took each action in the immediate aftermath are very similar across social contexts. Similarly, regarding RQ7 (Do actions taken during shaking affect behavior during the immediate aftermath?), activities during the immediate aftermath were only weakly related to behavior during the shaking. Those who froze were more likely to resume previous activities (*r* = 0.17) and those who tried to protect persons were more likely to help others (*r* = 0.18), but these were the only two significant correlations out of 30 total correlations between actions during the two time periods.

Regarding RQ8 (What communication channels were used to seek additional information about the situation and to contact separated household members?), people most frequently used telephone (60.8%), followed by face-to-face conversation (53.7%), Internet (47.3%, measured only in Wellington), radio (34.7%), and television (13.9%). To obtain information about the situation, people were far more likely to use the Internet (*r* = 0.49) than TV (*r* = 0.15) or radio (*r* = 0.19). To contact household members, however, they tended overwhelmingly to use telephones (*r* = 0.40) and Internet—presumably email (*r* = 0.22). Those who helped others quite logically tended to use face-to-face communication (*r* = 0.17). Finally, those who took the largest number of actions tended to use face-to-face communication (*r* = 0.17), radio (*r* = 0.18), phone (*r* = 0.26), and Internet (*r* = 0.39).

Partially consistent with RH1 (Perceived shaking intensity, risk perception and affective response are negatively correlated with returning to previous activities, but positively correlated with behaviors such as turning off utilities, contacting household members, cleaning up, and helping others), perceived shaking intensity was positively related to contacting household members (*r* = 0.18) and fear was positively related to seeking additional information (*r* = 0.18), contacting household members (*r* = 0.27), and helping others (*r* = 0.15). Finally, shock (*r* = 0.15) and fear (*r* = 0.29) were positively related to the total number of actions taken, fear was positively related to phone (*r* = .19), and all three affective variables were related to Internet (*r* = 0.23 for vigilance, 0.17 for shock, and 0.40 for fear). Risk perception was positively related to contacting household members (*r* = 0.22), helping others (*r* = 0.19), and Internet use (*r* = 0.41).

Contrary to RH2 (Home damage and infrastructure interruption are negatively correlated with returning to previous activities, but positively correlated with behaviors such as turning off utilities, contacting household members, cleaning up, and helping others), earthquake damage and infrastructure interruption had no significant impacts on people’s behavior in the immediate aftermath. Also contrary to expectations, the results for RH3 (Injuries are positively correlated with helping others but negatively correlated with the other immediate aftermath actions) indicate that injuries were not significantly correlated with immediate aftermath actions.

To better understand the variables that might be causally related to immediate aftermath behaviors, binary logistic regression analyses examined the effects of the most highly correlated predictor variables. One physical context variable (being in one’s own home) and one household context variable (member absent and safety unknown) were included as single items. Earthquake experience and emergency preparedness were standardized and added to produce an experience/preparedness index. In addition, perceived shaking intensity, shock, fear, and risk perception had moderately strong intercorrelations (average *r* = 0.34), so they were standardized and added to produce an index of psychological reactions. Similarly, home damage and infrastructure interruption were standardized and added to produce a damage index.

As Table 3 indicates, the regression of aftermath behaviors onto the experience/preparedness index produced three significant coefficients, indicating that the more prepared the participants were, the less likely they were to return to previous activities and the more likely they were to be involved with activities such as turning off utilities and helping others. In addition, physical context (whether the respondents were at home) produced three significant coefficients, indicating that people who were at home showed higher likelihood of returning to previous activities and cleaning up broken items but a lower likelihood of contacting household members. The latter result is a consequence of the respondents who were at home being more likely to be together with other household members. Household context (members absent and their safety unknown) had only one significant coefficient; respondents in this context were more likely to contact household members. The psychological reactions index (Psy Idx) produced five significant coefficients, with those having a stronger psychological reaction being less likely to return to previous activities, and more likely to seek additional information, contact household members, clean up debris, and help others. The physical damage index (Dmg Idx) generated one significant coefficient, negatively affecting resumption of previous activities. The dummy variable for Christchurch indicates that residents of this city were less likely than those in the other two cities to seek additional information about the situation. Finally, the dummy variable for Christchurch indicates that these residents were less likely than those in the other two cities to return to previous activities, seek additional information about the situation, contact household members, or help others. However, they were more likely to turn off utilities.

## 5. Discussion

The study extends the findings of previous research on immediate responses during earthquake shaking by examining actions in the earthquake’s immediate aftermath (the first 30 min after shaking stopped). In addition, it examined the effects of demographic variables; earthquake experience and emergency preparedness; physical, household and social context; and actions taken during shaking on actions in the earthquakes’ immediate aftermath. Finally, it examined the effects of psychological variables (perceived shaking intensity, risk perception, and affective response), injuries, damage and infrastructure disruption on actions in the immediate aftermath. The present study found a number of similarities to Takuma’s [20] data on people’s actions after the 1968 Ebino earthquake. Specifically, compared to the Ebino sample, the Christchurch and Hitachi samples had similar levels of returning to previous activities (7% vs. 13% and 6%, respectively), turning off utilities (18% vs. 18% and 20%, respectively), and seeking shelter (24% vs. 30% and 27%, respectively). However, there were major differences in seeking information (8% vs. 37% and 33%, respectively), and cleaning up debris (5% vs. 30% and 30%, respectively). The Lake Grassmere and Cook Strait samples tended to have even larger differences, very likely due to their lower shaking intensities and durations.

Perhaps the most noteworthy finding is that actions in the immediate aftermath have trivial correlations with responses during earthquake shaking—average *r* = 0.02. In some respects, this is unsurprising because responses during earthquake shaking have trivial average correlations with all of the other groups of variables—demographic (average *r* = 0.00), experience/preparedness (average *r* = 0.04), contextual (average *r* = 0.02), and psychological (average *r* = 0.09). Actions in the immediate aftermath also have trivial average correlations with most groups of variables—demographic (average *r* = 0.02), experience/preparedness (average *r* = 0.08), context (average *r* = 0.02), and injury (average *r* = 0.03), but they have significant average correlations with the psychological variables (average *r* = 0.17) and damage (average *r* = 0.15) when the signs of the correlations for resuming previous activities are reversed (because this is expected to be correlated with lower risk perception and damage). It is difficult to make direct comparisons between the results of the present study and those from previous studies because, as noted earlier, it appears that few studies have examined response actions immediately following earthquake shaking. Thus, the present results will be compared directly to those of previous studies with regard to immediate responses during shaking, and the relevance for actions within the first 30 min after shaking will be drawn from those comparisons. Another major question of this study concerns whether actions during shaking and the immediate aftermath were differentially correlated with some potential predictor variables (e.g., demographic, experience/preparedness, and contextual variables, as well as perceived shaking intensity, risk perception, and affective response).

As for RQ1 (How did behavior during the immediate aftermath differ across earthquakes?), the results showed that, compared to the 2013 Wellington earthquakes, the 2011 earthquakes (Christchurch and Hitachi) had higher percentages of people who turned off utilities and cleaned up broken or fallen items and lower percentages of people who returned to their previous activities. On the other hand, the 2013 Wellington earthquakes produced higher percentages of people who sought information after the shaking stopped, as compared to 2011 earthquakes in Christchurch and Hitachi. These differences may be due to the greater shaking intensity during the Christchurch and Hitachi earthquakes (and especially the greater shaking duration in Hitachi), which would have generated a greater need for people to turn off utilities or clean up debris. Those affected by the former two earthquakes might have been too busy responding to their immediate situations to be concerned about seeking information. In addition, many more people in Hitachi and Christchurch than in Wellington experienced interruption of electric power (99%, 88%, and 2%, respectively), TV (87%, 46%, and 2%, respectively), telephone (83%, 67%, and 5%, respectively), and only 3% of those in Wellington experienced interruption of the Internet. Thus, the lower levels of information seeking in Hitachi and Christchurch is probably due, at least partially, to lack of access.

Concerning RQ2 (Do demographic variables affect behavior during the immediate aftermath?), the results reveal that gender was uncorrelated with immediate aftermath actions, whereas Bourque et al. [16] reported that female gender was negatively correlated with immediate protective actions during shaking. Because the present study combined all others as a single referent and did not differentiate family members from peers (friend, relatives, neighbors, or coworkers) or strangers, it is difficult to make a direct comparison between the present results and those of Form and Nosow [24], who reported that females were less likely to render help to strangers. Moreover, the present results indicate older people were less likely to be involved in contacting household members (*r* = −0.25), or seeking information (*r* = −0.11), but were more likely to turn off utilities (*r* = 0.11). These results are somewhat different from Friedsam’s [23] findings, which found that helping others beyond family was negatively related to age. Here, too, it is difficult to draw a conclusive comparison due to the differences in question wording.

Income also appears to have a modest effect on immediate aftermath actions; participants with higher incomes were less likely to turn off utilities (*r* = −0.12) and more likely to contact household members (*r* = 0.14). However, education and marital status have minimal effects on immediate aftermath actions; higher levels of education increase the search for additional information about the situation (*r* = 0.11) and marital status increases the likelihood of contacting household members (*r* = 0.11). Overall, these results indicate that demographic variables have quite modest effects on immediate aftermath actions.

RQ3 (Do earthquake experience and emergency preparedness affect behavior during the immediate aftermath?) addressed whether the participants’ personal experiences with earthquakes and their subsequent awareness/preparedness predict immediate aftermath behaviors. This study’s results indicate that respondents who had received earthquake information prior to their earthquakes were more likely to turn off utilities (*r* = 0.12) and help others (*r* = 0.11). These results could be related with previous findings that reported a positive effect of previous experience and preparedness on appropriate immediate responses to extreme events [13]. However, the results of the present study are slightly different in that, although earthquake information did not have significant correlations with immediate responses during shaking, it did seem to have positive impacts on behaviors after shaking stopped. This result suggests that current disaster education and information programs are at least partially effective. They seem to have a significant impact on response actions after shaking stops even though not on response actions during shaking.

With regard to RQ4 (Does physical context affect behavior during the immediate aftermath?), it is noteworthy that people who were at home at the time of earthquake were less likely to contact household members than those who were elsewhere (*r* = −0.19). It should be noted, however, that those who were at home were very likely to be with other household members (*r* = 0.75). Therefore, this result should be interpreted with the impacts of household context (RQ5), which addressed whether being together with household members affected people’s immediate aftermath behaviors. It seems that being at home, where participants were likely to be with their families, largely eliminated uncertainty about the safety of other household members but this did not help them to return to their normal activities. Moreover, the results regarding RQ5 (Does household context affect behavior during the immediate aftermath?) revealed that participants were more likely to contact absent household members if they were unsure of the absent members’ safety (*r* = 0.23), or even if they were known to be safe (*r* = 0.17). By contrast, those who were safely together with household members were less likely to make contact (*r* = −0.30) and were more likely to turn off utilities (*r* = 0.11). These results suggest that knowing the safety of family members can allow people to take other actions during the immediate aftermath but the effect is very weak. This is consistent with the finding that household context did not show significant correlations with immediate response actions.

The results for RQ6 (Does social context affect behavior during the immediate aftermath?) revealed that people’s social context (whether they were alone, with children, or with adults) produced differences in immediate aftermath actions. Consistent with previous studies, those who were with children were more likely to help others (*r* = 0.12), whereas those who were alone were less likely to do so (*r* = −0.13). These results are somewhat consistent with Goltz et al. [17], who reported that the presence of other adults inhibited protective action, whereas the presence of children enhanced it. These results could also be compared with previous studies where contextual variables had limited impacts on immediate behavioral responses during shaking [19,36]; these results indicate that social context, like household context, has a modest effect on immediate aftermath actions.

Regarding RQ7 (Do actions taken during shaking affect behavior during the immediate aftermath?), the results revealed that actions during and after earthquake shaking had only two significant correlations: those who froze during shaking were more likely to return to previous activities (*r* = 0.17) and those who tried to protect persons during shaking were more likely to help others afterward (*r* = 0.18). Both findings indicate a continuity between actions during shaking and immediately afterward. As for RQ8 (What communication channels were used to seek additional information about the situation and to contact separated household members?), the Internet showed the strongest correlation with information-seeking behavior (*r* = 0.49; data collected only in Wellington), although there were significant correlations for the other communication channels as well. By contrast, telephone seems to have been the major communication channel for contacting household members (*r* = 0.40) although the Internet (*r* = 0.22) and face-to-face communication (*r* = 0.11) also received significant use. The results concerning the Internet are consistent with recent conclusions about its increasing importance as a source of information and risk communication [37,38] compared to the low reliance on the Internet that previous research has found during events that occurred in earlier years [36,39].

The partial support for RH1 (Perceived shaking intensity, risk perception and affective response are negatively correlated with returning to previous activities, but positively correlated with behaviors such as turning off utilities, contacting household members, cleaning up, and helping others) indicates that the higher people’s perceived shaking intensity and risk perceptions, the less likely they were to resume their previous activities and the more likely they were to engage in actions that responded to the shaking. These correlations ranged from weak (*r* = 0.15) to moderately strong (*r* = 0.32). These results are interesting when considered in relation to the correlations between these psychological variables and the immediate responses during the shaking, which were nonsignificant. These findings are consistent with those reported by Ohta and Ohashi [18], who reported that efforts to “tidy rooms” and “purchase essentials” increased with shaking intensity (from just over 1% to almost 50% of respondents), whereas information search by TV or radio was constant (at about 50%) and return to previous activities decreased with shaking intensity (from about 40% to just over 1%). These results provide an important qualification to previous conclusions that high perceived shaking intensity decreases some people’s adaptive response during an earthquake. Specifically, Takuma [20] reported that more people evacuated immediately (39%) than any other action and Lindell et al. [13] found that perceived shaking intensity was correlated with immediate evacuation. These results suggest that people become increasingly likely to engage in adaptive behavior after the immediate threat passes.

In order to explore the impacts of physical damage and injuries on immediate aftermath actions, RH2 (Home damage and infrastructure interruption are negatively correlated with returning to previous activities, but positively correlated with behaviors such as turning off utilities, contacting household members, cleaning up, and helping others) and RH3 (Injuries are positively correlated with helping others but negatively correlated with the other immediate aftermath actions) were tested. The weak support for RH2 showed that the more damage people experienced, the more likely they were to clean up debris in the immediate aftermath (*r* = 0.11). Surprisingly, injuries were nonsignificantly correlated with immediate aftermath actions, perhaps because the injuries these respondents experienced were rare and might have been quite minor—slight cuts and bruises rather than major lacerations and fractures.

## 6. Conclusions

In summary, there are two key findings from this study. First, household context, especially knowing the safety of absent household members, had a moderately strong relation to people’s immediate aftermath actions; the most common immediate aftermath action was contacting family members (54%). These results indicate that more planning/policy efforts should be made to encourage households to develop plans for communicating with family members when away from the home as well as to facilitate communication among household members in the earthquake aftermath. Both efforts will reduce people’s psychological stress and facilitate an earlier recovery from the disaster.

Second, there was only a modest continuity between actions taken during earthquake shaking and those taken during the immediate aftermath; the only two significant correlations were for freezing during shaking with returning to previous activities afterward and protecting persons during shaking and helping others afterward. Moreover, the antecedent demographic, contextual, and psychological variables had only 11 significant correlations with actions during shaking but 29 significant correlations with immediate aftermath actions. This difference in the predictability of earthquake response during the two time periods poses an important challenge for future research, specifically whether there are other variables that can provide better prediction of actions during shaking. A related concern is that earthquake experience, earthquake information, and emergency preparedness had no significant correlations with actions during shaking and only two small correlations with immediate aftermath action. The significant correlation and regression coefficients for turning off utilities suggests that current earthquake information and preparedness have at least one specific effect on people’s earthquake response actions.

However, the nonsignificant correlations of earthquake experience, information, and preparedness with actions during shaking suggest that earthquake experience did not teach people what to do in subsequent earthquakes and the available earthquake information and preparedness checklists—if they did address this information—did not provide it in a way that led people to take appropriate action when it was needed during seismic shaking. This suggests that existing hazard awareness programs may need to be examined to see if they can be revised in ways that facilitate transfer of training [40,41] from the “classroom” to actual events so people know—and remember—how to respond appropriately during shaking and have such confidence in this training that they have lower levels of risk perception and affective response.

One promising aspect of these results is that they identify promising avenues for emergency managers to communicate seismic risks and appropriate responses to risk area populations. Specifically, the correlation and regression coefficients for the psychological reactions are important because they indicate that these variables contribute significantly to the prediction of all immediate aftermath actions other than turning off utilities. In turn, the psychological reactions are significantly higher among women and younger respondents. Some of these results are quite consistent with previous research, especially the higher level of psychological reactions among women [42], which has been explained as effects of lower socioeconomic status [43]. The correlations of these demographic variables suggest a strategy of audience segmentation [25] (p. 192), in which emergency managers reach out to specific population segments with information about appropriate earthquake preparedness and response actions.

One limitation of the present study is that it relies on self-report data about people’s actions during and immediately after earthquake shaking. Future research should supplement post-impact survey data with the analysis of behavioral responses during and immediately after earthquake shaking that are captured on closed-circuit television (CCTV) recordings [44]. Such data necessarily have limitations such as paucity of locations (mostly public), limited field of view, lack of audio data, and data loss due to power outages. Nonetheless, CCTV data on behavioral responses in public locations could be systematically compared to corresponding survey data from such locations to determine if different research methods produce convergent results. A related limitation of these surveys is that they rely on self-reports of psychological states that were collected months after the events. This is a cause for some concern but not alarm because previous studies have found that people’s memories for dramatic events are stable over extended periods of time (see Lindell et al. [13,45], for discussion).

A second limitation is the modest response rates, which raises questions about the representativeness of the samples. In fact, like many mail surveys, the sample over-represents older, married, home-owning, and more highly educated respondents. It is noteworthy that over-representation of some demographic categories will produce bias in other variables, such as risk perception and hazard adjustment, only to the degree the latter variables are correlated with demographic variables. Such correlations, however, are low in this sample, as well as more generally [6,7,16,46]. Moreover, low response rates do not appear to bias central tendency estimates such as means and proportions [7,47,48]. Additionally, there are psychometric reasons for believing low response rates are unlikely to affect correlations [7], and analysis of the immediate response data from Christchurch and Hitachi suggested that nonresponse did, indeed, produce minimal bias in the estimation of the correlation coefficients [13].

Finally, although this study appears to be unique in reporting data from four different earthquakes, three of them were from a single country and the fourth was from an event that had a substantially greater magnitude than the others. This concern is supported by the significant regression coefficients for the Hitachi dummy code in the regression analysis of all immediate aftermath actions; except for cleaning up debris, the dummy code suggests that there are some important unmeasured variables that differentiate the response to this earthquake from the responses to the New Zealand earthquakes. Thus, future studies need to investigate a broader range of countries when examining the effects of demographic, experiential, and contextual variables on psychological reactions, actions taken during seismic shaking, and immediate aftermath actions. As in the present study, this will raise the possibility that some of the differences between sites in people’s responses might be due to unmeasured variables such as differences in shaking duration, structural seismic resilience, and household seismic preparedness (e.g., previously installing cupboard latches and bolting tall appliances to the walls). However, such systematic comparisons among earthquakes are needed to advance the scientific understanding of people’s behavior in such events.

## Figures and Tables

**Table 1:** Demographic characteristics of each site, compared to Census data.

Demographic	Sample			Census		
Variables	Christchurch	Hitachi	Wellington	Christchurch	Hitachi	Wellington
Female Gender %	65%	42%	48%	51%	50%	52%
Mean Education Level	3.1	3.6	3.6	n/a	n/a	n/a
Mean Income Level	$48,000	$29,000	$42,000	$20,630	$29,688*	$27,434
Married %	57%	85%	50%	45%	46%	46%
Mean Age	52.8	62.0	49.6	39.0	51.6	35.3
Home Ownership %	79%	90%	80%	52%	62%	55%

Note: In Christchurch and Wellington, the respondent’s education level was categorized: no school qualifications; school qualifications such as trade certificate, professional certificate, or diploma; university undergraduate degree (such as university diploma or bachelor’s degree); or university postgraduate degree (such as a Master’s or a Ph.D.). In Hitachi, the response options were: less than ninth grade; ninth to twelfth grade, no diploma; high-school graduate (includes equivalency); some college, no degree; associate degree; bachelor’s degree; or graduate or professional degree. Yearly household income was categorized as less than USD 20,000, between USD 20,000 and 29,999, USD 30,000 and 39,999, or USD 40,000 and 49,999, or USD 50,000 or more, and was translated into the corresponding currency (NZD or JPY) at the prevailing exchange rate. Hitachi income is the average gross income per person. n/a = not available.

**Table 2:** Means (M), Standard deviations (SD), and intercorrelations among variables.

**Variable**	**M**	**SD**	**1**			**2**	**3**	**4**	**5**	**6**	**7**	**8**	**9**	**10**	**11**	**12**	**13**	**14**	**15**	**16**	**17**	**18**
1. Female	0.51	0.50																				
2. Age	54.49	15.72	−0.09																			
3. Education	3.48	1.32	0.00			−0.28																
4. Income	3.38	1.71	−0.08			−0.41	0.40															
5. Married	0.63	0.48	−0.13			−0.03	0.07	0.25														
6. EQ_Exp	0.13	0.21	−0.04			0.05	0.04	0.04	−0.03													
7. EQ_Info	0.27	0.32	0.05			0.12	0.02	0.05	0.03	0.20												
8. EmergPrep	0.26	0.30	0.05			0.05	−0.09	−0.05	0.01	0.07	0.17											
9. PC_Home	0.33	0.47	−0.06			0.27	−0.15	−0.25	−0.05	0.00	−0.03	0.09										
10. HC_Together	0.28	0.45	−0.10			0.28	−0.14	−0.25	−0.08	0.02	0.00	0.08	0.75									
11. HC_AbSafe	0.21	0.41	−0.01			−0.10	−0.03	0.04	0.06	−0.02	0.03	0.08	−0.17	−0.32								
12. HC_AbUnk	0.41	0.49	0.06			−0.25	0.18	0.26	0.07	0.02	−0.01	−0.12	−0.39	−0.49	−0.45							
13. SC_Alone	0.29	0.46	0.00			0.21	−0.12	−0.19	−0.15	0.00	−0.07	−0.03	0.10	−0.07	0.06	0.01						
14. SC_Child	0.14	0.35	0.13			−0.16	0.03	0.07	0.11	0.03	0.02	0.04	0.02	0.02	0.03	0.00	−0.26					
15. SC_Adult	0.61	0.49	−0.04			−0.13	0.07	0.13	0.09	−0.02	0.09	0.03	−0.03	0.12	−0.01	0.01	−0.77	−0.09				
16. EQFelt	4.74	1.53	0.04			−0.09	0.04	0.03	−0.02	0.01	−0.02	0.06	0.10	0.01	0.04	0.09	0.02	0.05	0.14			
17. Aff_Vigil	1.53	1.03	−0.13			−0.07	0.00	0.04	0.07	0.01	−0.01	0.01	0.09	0.05	0.10	−0.03	0.01	0.03	0.04	0.00		
18. Aff_Shock	1.53	1.30	−0.01			0.00	−0.05	−0.01	0.00	0.04	0.08	0.02	0.07	0.02	0.12	−0.03	0.06	−0.02	−0.04	0.12	0.20	
19. Aff_Fear	3.39	1.43	0.15			−0.23	0.06	0.05	0.02	0.01	0.01	−0.01	0.03	−0.04	0.06	0.09	0.02	0.09	0.03	0.42	0.02	0.36
20. RiskPer	2.47	1.39	0.06			−0.18	0.06	0.10	0.01	0.06	0.05	0.08	−0.04	−0.09	0.04	0.12	−0.01	0.02	0.08	0.36	0.03	0.29
21. Im_Freeze	0.32	0.47	−0.06			0.10	−0.08	−0.06	−0.02	−0.03	−0.03	−0.01	0.11	0.07	0.07	−0.06	0.10	−0.11	0.00	−0.02	0.03	0.06
22. Im_DropCov	0.16	0.36	0.01			−0.07	0.04	0.10	0.05	0.08	0.06	0.06	−0.05	−0.01	−0.02	0.07	−0.10	−0.04	0.15	0.13	−0.05	0.01
23. Im_ProtPers	0.07	0.26	0.06			−0.13	0.00	0.04	0.04	0.00	0.01	0.01	0.03	0.01	0.00	−0.01	−0.17	0.42	0.02	0.05	0.09	0.00
24. Im_ProtProp	0.05	0.22	0.00			0.06	−0.02	−0.03	−0.01	0.00	0.01	−0.03	0.07	0.03	0.00	−0.05	0.08	−0.07	−0.05	0.01	0.00	0.02
25. Im_Evacuate	0.22	0.42	0.05			−0.03	0.03	−0.01	−0.03	−0.01	−0.01	0.01	0.05	−0.02	0.00	0.05	0.01	−0.02	0.02	0.16	0.02	0.03
26. HH_MemInj	0.16	0.37	0.04			−0.14	0.00	0.00	−0.01	−0.01	0.02	0.08	0.02	−0.01	−0.02	0.09	0.00	0.03	0.04	0.13	0.02	0.02
27. HomeDmg	1.93	0.91	0.14			0.00	0.04	0.02	0.00	0.16	0.02	0.01	−0.03	−0.05	−0.01	0.03	0.00	0.10	−0.03	0.10	0.01	0.04
28. InfraInter	2.59	2.51	0.09			−0.09	0.08	0.04	0.02	0.08	0.05	0.12	−0.07	−0.05	−0.07	0.09	0.04	0.03	−0.02	0.09	−0.07	0.00
29. 30m_Return	0.25	0.43	−0.06			0.02	−0.01	−0.05	−0.04	−0.04	−0.03	−0.05	0.07	0.06	0.01	0.02	0.03	−0.04	0.06	0.05	0.09	0.01
30. 30m_SeekInf	0.42	0.49	0.07			−0.11	0.11	0.05	0.06	0.01	0.05	0.06	0.10	0.04	0.07	−0.03	0.04	0.07	0.02	0.10	0.06	0.08
31. 30m_TurnOff	0.12	0.33	0.03			0.11	−0.08	−0.12	−0.05	0.10	0.12	0.07	0.09	0.11	−0.02	−0.07	0.07	−0.02	−0.04	−0.01	0.01	0.04
32. 30m_Contact	0.54	0.50	0.09			−0.25	0.01	0.14	0.11	0.04	−0.03	0.03	−0.19	−0.30	0.17	0.23	0.03	0.02	0.02	0.18	0.03	0.11
33. 30m_Cleanup	0.23	0.42	0.01			0.04	−0.09	−0.07	−0.07	0.03	0.07	0.07	0.10	0.07	0.03	−0.06	0.10	−0.01	−0.05	0.08	0.04	0.13
34. 30m_HlpOth	0.19	0.39	0.07			−0.07	0.05	0.08	−0.01	0.04	0.11	0.07	−0.02	−0.06	0.00	0.07	−0.13	0.12	0.10	0.13	0.09	0.09
35. 30m_Count	2.29	1.31	0.08			−0.18	0.05	0.09	0.02	0.08	0.08	0.10	−0.05	−0.11	0.14	0.09	0.04	0.04	0.07	0.22	0.13	0.15
36. Chan_Face	0.54	0.50	0.10			−0.17	0.16	0.13	0.06	−0.01	0.05	0.02	−0.05	0.00	0.01	0.06	−0.15	0.05	0.21	0.17	0.11	0.05
37. Chan_TV	0.14	0.35	−0.04			0.05	−0.04	−0.01	0.07	−0.02	0.05	0.04	0.18	0.08	0.10	−0.13	0.05	0.09	−0.01	0.03	0.03	0.12
38. Chan_Radio	0.35	0.48	−0.04			0.08	−0.08	−0.10	0.01	0.01	0.02	0.10	0.11	0.11	0.07	−0.12	0.08	0.00	0.00	0.08	0.06	0.10
39. Chan_Phone	0.61	0.49	0.00			−0.23	0.08	0.21	0.12	0.02	−0.05	0.00	−0.07	−0.15	0.10	0.19	−0.04	−0.02	0.12	0.18	0.07	0.09
40. Chan_Intrnet	0.47	0.50	0.09			−0.35	0.23	0.25	0.07	0.02	0.08	0.05	0.14	0.05	0.04	0.12	−0.05	0.12	0.16	0.22	0.23	0.17
41. Christchurch	0.26	0.44	0.16			−0.07	−0.19	0.1	−0.07	0.67	0.10	0.51	−0.09	−0.03	−0.04	0.08	0.01	0.01	0.02	0.33	0.18	0.09
42. Hitachi	0.33	0.47	−0.40			0.33	0.08	−0.57	0.32	−0.20	−0.32	0.02	−0.21	−0.14	0.03	0.19	0.03	−0.13	0.07	0.48	0.06	0.62
43. Cook Strait	0.20	0.40	0.15			−0.16	0.05	0.25	−0.14	−0.25	0.13	−0.29	0.37	0.29	−0.03	−0.28	0.03	0.14	−0.11	−0.48	−0.16	−0.41
44. L. Grassmere	0.20	0.40	0.15			−0.16	0.05	0.25	−0.14	−0.25	0.13	−0.29	−0.02	−0.09	0.04	−0.03	−0.09	0.00	0.00	−0.43	−01	−0.36
**Variable**	**19**	**20**	**21**	**22**	**23**	**24**	**25**	**26**	**27**	**28**	**29**	**30**	**31**	**32**	**33**	**34**	**35**	**36**	**37**	**38**	**39**	**40**
20. RiskPer	0.51																					
21. Im_Freeze	−0.10	−0.12																				
22. Im_DropCov	0.14	0.10	−0.28																			
23. Im_ProtPers	0.09	0.03	−0.19	−0.11																		
24. Im_ProtProp	0.01	−0.02	−0.15	−0.07	−0.06																	
25. Im_Evacuate	0.15	0.18	−0.36	−0.21	−0.14	−0.11																
26. HH_MemInj	0.14	0.18	−0.04	0.07	−0.02	0.02	0.04															
27. HomeDmg	0.10	0.18	−0.07	0.02	0.01	0.02	0.05	0.03														
28. InfraInter	0.06	0.12	0.02	0.03	−0.03	0.01	0.05	0.09	0.17													
29. 30m_Return	−0.02	−0.05	0.17	−0.08	−0.03	−0.02	−0.03	−0.01	−0.07	−0.04												
30. 30m_SeekInf	0.18	0.13	0.01	0.06	0.04	0.01	0.01	0.07	0.01	0.06	0.01											
31. 30m_TurnOff	0.02	0.14	−0.04	0.01	0.02	−0.02	0.08	0.05	0.03	0.05	−0.10	0.08										
32. 30m_Contact	0.27	0.22	0.02	0.04	0.01	−0.02	0.06	0.08	0.02	0.10	0.00	0.19	0.04									
33. 30m_Cleanup	0.12	0.13	−0.04	0.03	0.02	0.07	0.04	−0.03	0.11	0.05	−0.01	0.07	0.23	0.08								
34. 30m_HlpOth	0.15	0.19	−0.06	0.04	0.18	−0.03	−0.01	0.06	0.04	0.02	−0.07	0.09	0.04	0.14	0.11							
35. 30m_Count	0.29	0.28	−0.01	0.05	0.05	−0.06	0.09	0.09	0.08	0.13	0.19	0.49	0.35	0.58	0.41	0.38						
36. Chan_Face	0.09	0.09	−0.07	0.09	0.02	−0.05	0.06	0.04	0.05	0.07	−0.01	0.13	0.05	0.11	0.06	0.17	0.22					
37. Chan_TV	0.08	0.04	0.02	−0.02	0.05	−0.02	0.07	0.03	−0.06	−0.05	0.04	0.15	0.08	0.00	0.12	0.00	0.09	−0.01				
38. Chan_Radio	0.07	0.05	0.02	−0.04	0.05	0.02	−0.01	0.04	−0.01	−0.01	−0.05	0.19	0.11	0.08	0.12	0.05	0.18	0.03	0.07			
39. Chan_Phone	0.19	0.15	0.07	0.11	−0.02	−0.06	0.02	0.07	0.03	0.00	0.07	0.12	−0.04	0.40	0.05	0.06	0.26	0.15	0.04	0.06		
40. Chan_Intrnet	0.40	0.41	−0.04	0.08	0.07	−0.01	0.17	0.13	0.07	0.04	0.09	0.49	−0.09	0.22	0.16	0.15	0.39	0.20	0.19	0.08	0.26	
41. Christchurch	0.04	0.15	0.07	0.03	0.07	0.00	−0.17	−0.18	0.45	0.27	−0.17	−0.06	0.10	0.15	0.10	0.33	0.26	0.10	−0.12	0.15	0.16	−−
42. Hitachi	0.46	0.53	−0.01	−0.16	−0.02	0.16	0.09	−0.28	0.22	0.62	−0.31	−0.14	0.16	0.03	0.12	−0.20	−0.07	−0.06	0.00	0.03	−0.22	−−
43. Cook Strait	−0.30	−0.44	−0.02	−0.09	0.01	−0.10	0.14	0.28	−0.38	−0.50	0.34	0.10	−0.12	−0.17	−0.09	−0.12	−0.14	−0.07	0.15	−0.07	−0.01	−0.06
44. L. Grassmere	−0.25	−0.34	−0.05	0.25	−0.06	−0.09	−0.07	0.23	−0.40	−0.51	0.21	0.12	−0.17	−0.03	−0.16	−0.01	−0.05	0.03	−0.03	−0.13*	0.09	0.06

Notes: EQ_Exp = previous earthquake experience, EQ_Info = previous earthquake information, EmergPrep = number of prior emergency preparedness actions, PC_Home = physical context: in own home, HC_Together = all household members together, HC_AbSafe = some household members absent/known safe, HC_AbUnk = some household members absent/safety unknown, SC_Alone = social context: alone, SC_Child = social context: with children, SC_Adult = social context: with adults, EQFelt = perceived shaking intensity, Aff_Vigil = affective reaction: vigilance, Aff_Shock = affective reaction: shock, Aff_Fear = affective reaction: fear, RiskPer = risk perception, Im_Freeze = immediate response: freeze, Im_DropCov = immediate response: Drop/Cover, Im_ProPers =immediate response: protect persons, Im_ProProp = immediate response: protect property, Im_Evac = immediate response: evacuation, HH_MemInj = had household members injured from earthquake, HomeDmg = had home damage, InfraInter = number of infrastructure interrupted, 30m_Return = aftermath behavior: return to previous activity, 30m_SeekInfo = aftermath behavior: seek further information, 30m_TurnOff = aftermath behavior: turn off utilities, 30m_Contact = aftermath behavior: contact household members, 30m_Cleanup = aftermath behavior: clean up broken items, 30m_HlpOthr = aftermath behavior: help others, 30m_Count = aftermath behavior: number of aftermath behaviors taken, Chan_Face = communication channel: face to face, Chan_TV = communication channel: TV, Chan_Radio = communication channel: radio, Chan_Phone = communication channel: phone, Chan_Intrnet = communication channel: internet (measured only in Wellington communities for the Cook Strait and Lake Grassmere earthquakes). Total N = 997, *p*(r_ij_ > 0.11) < 0.001; Christchurch N = 257, *p*(r_ij_ > 0.16) < 0.01; Hitachi N = 332, *p*(r_ij_ > 0.14) < 0.01, Cook Strait and Lake Grassmere N = 204, *p*(r_ij_ > 0.18) < 0.01.

**Table 3:** Regression results.

Predictor	Dependent Variable					
Variables	Return	Seek Info	Turn off	Contact	Clean up	Help Others
Exp Prep Idx	−0.17 *	0.06	0.20 *	0.10	0.04	0.15 *
	(0.08)	(0.06)	(0.09)	(0.07)	(0.07)	(0.07)
Own Home	0.37 *	0.25	0.45	−0.97 *	0.62 *	−0.39
	(0.20)	(0.17)	(0.30)	(0.18)	(0.22)	(0.24)
HC AbUnk	−0.16	−0.18	−0.13	0.57 *	−0.29	0.38 *
	(0.21)	(0.16)	(0.26)	(0.17)	(0.20)	(0.21)
Psy Idx	−0.08 *	0.15 *	0.09	0.25 *	0.24 *	0.32 *
	(0.04)	(0.03)	(0.06)	(0.04)	(0.05)	(0.05)
Dmg Idx	−0.31 *	−0.00	0.10	−0.09	0.02	0.00
	(0.11)	(0.07)	(0.10)	(0.08)	(0.08)	(0.09)
Christchurch	−0.29	−1.38 *	0.81	−0.25	0.28	−0.22
	(0.43)	(0.35)	(0.54)	(0.37)	(0.40)	(0.41)
Hitachi	−1.26 *	−1.65 *	1.12 *	−1.31 *	−0.06	−2.67 *
	(0.47)	(0.33)	(0.53)	(0.36)	(0.38)	(0.44)
Constant	−0.96 *	0.63 *	−3.04 *	0.86 *	−1.55 *	−0.91 *
	(0.29)	(0.23)	(0.42)	(0.24)	(0.28)	(0.286)
Observations	846	846	846	845	846	846
Log Likelihood	−378.95	−552.20	−274.30	−506.35	−417.80	−351.39
Nagelkerke R^2^	0.46	0.31	0.42	0.34	0.32	0.38

Note: "Exp Prep Idx" = preparedness/experience index (composite variable consisted of earthquake preparedness and previous experience), Own Home = physical context: in own home, HC AbUnk = some household members absent/safety unknown, "Psy Idx" = psychological index (composite variable consisted of perceived shaking intensity, shock, fear, and risk perception), "Dmg Idx" = damage index (composite variable consisted of home damage level and different infrastructure interruptions). *S*tandard errors in parentheses; * *p* < 0.05.
